# Stent retriever thrombectomy in the treatment of basal artery stent thrombosis: Two case reports

**DOI:** 10.1097/MD.0000000000030541

**Published:** 2022-09-09

**Authors:** Hang Li, Qinghai Dai, Lingfeng Shu, Tao Wu, Dongyi Yang, Yangyang Liu

**Affiliations:** a The First Clinical Medical College, Henan University of Traditional Chinese Medicine, Zhengzhou, Henan, China; b Department of Intervention, the First Affiliated Hospital of Henan University of Traditional Chinese Medicine, Zhengzhou, Henan, China.

**Keywords:** cerebrovascular stent, complication, ischemic stroke, stent retriever thrombectomy, stent thrombosis

## Abstract

Stent thrombosis is a rare but serious complication that requires timely treatment. Catheter thrombectomy is the first-line therapy for this condition; however, it may be challenging for patients with in-stent emboli located in the basilar artery.

**Patient concerns:**

Herein, we reported 2 cases with stent thrombosis in the vertebrobasilar artery, including their imaging and clinical findings. Both patients were successfully treated with stent retriever thrombectomy.

**Diagnosis:**

The presence of cerebral infarction was diagnosed by magnetic resonance. Cranial computed tomography excluded cerebral hemorrhage, and basilar artery occlusion was considered in combination with the medical history.

**Interventions:**

After cerebral angiography confirmed basilar artery occlusion, the stent thrombectomy was used to remove the thrombus.

**Outcomes:**

The emboli were removed from the stent, and the angiography confirmed that the blood flow in the posterior cerebral circulation was recovered to TICI level 3. Moreover, all symptoms disappeared.

**Lessons:**

Cerebral vascular stent thrombectomy is a feasible approach for treating cerebral vascular stent thrombosis.

## 1. Introduction

Cerebrovascular artery stenosis is an important factor associated with ischemic stroke.^[1]^ Over the years, stents have been used to recanalize occluded or severely stenosed cervical arteries in order to increase blood flow to the cerebral arteries in acute stroke patients who did not respond well to medical treatment or other treatments.^[2]^ However, this approach is associated with certain postoperative complications, including intracranial hemorrhage, stent thrombosis, and vasospasm.

Stent thrombosis is a rare but serious complication that requires timely treatment.^[3]^ A recent study reported a stent thrombosis rate of about 4% in patients receiving stent implantation.^[4]^ Herein, we reported 2 cases with stent thrombosis in the vertebrobasilar artery, including their imaging and clinical findings, who were successfully treated with stent retriever thrombectomy.

## 2. Case presentation

### 2.1. Case 1

A 62-year-old male with a history of type-2 diabetes suffered from dizziness that lasted for a month. While he did not have nausea, vomiting, dizziness, and loss of consciousness during the attack, he would repeatedly experience these symptoms after medical treatment. Magnetic resonance (MR) angiography showed the left and right vertebrobasilar artery stenosis, after which balloon dilation angioplasty (BDA) and stent implantation of the left vertebrobasilar artery, BDA, and stent implantation of the right vertebral artery were performed. Tirofiban 10 mL/h microinjection pump (ivvp) for 24 hours, aspirin 100 mg peros (po), clopidogrel 75 mg po were prescribed, after which dizziness and residual neurological dysfunction disappeared.

**Figure 1.**
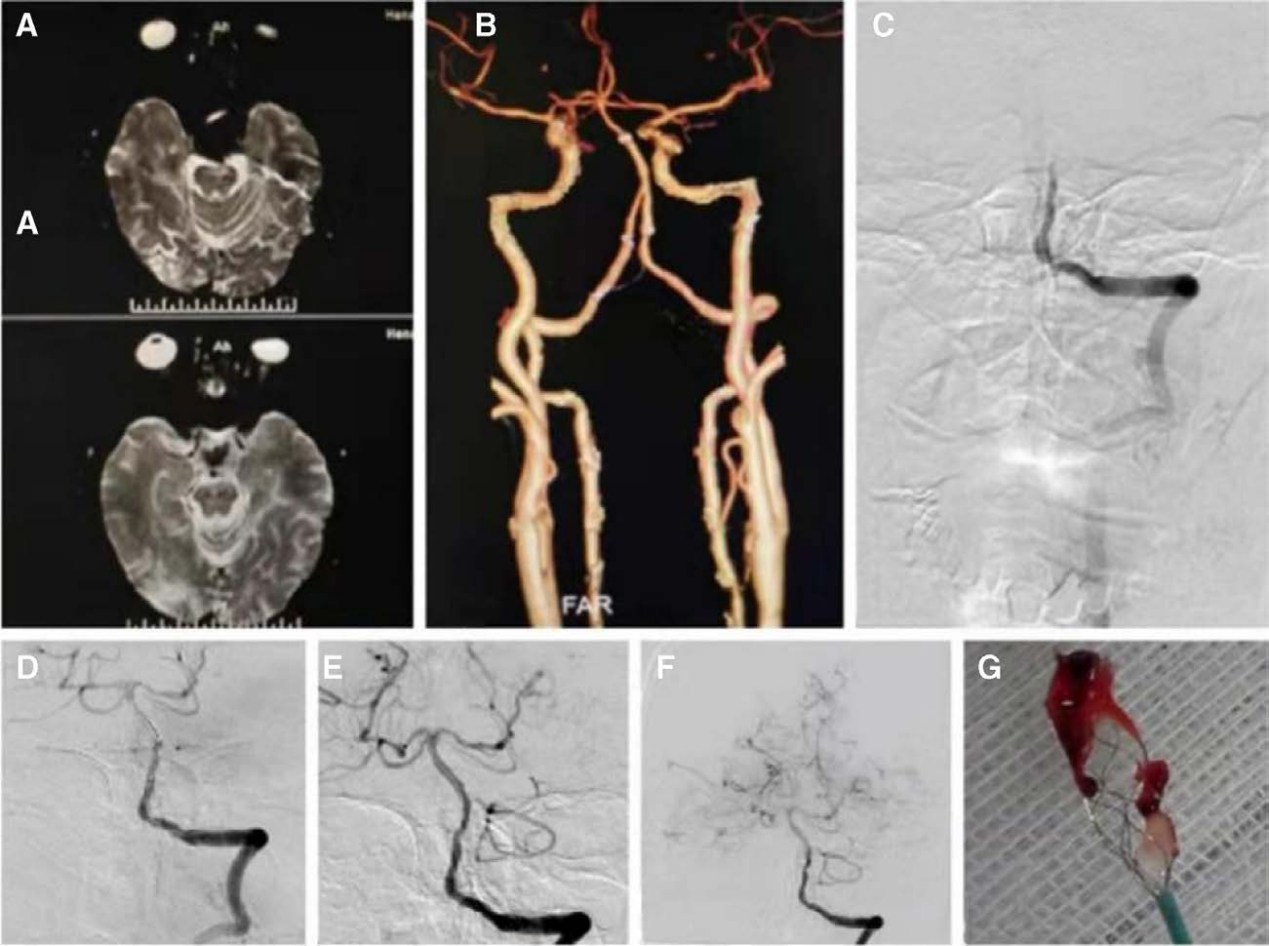
(A) DWI showing ischemic lesions in the left cerebral peduncles. (B) CTA showing basilar artery stenosis. (C) Basilar artery occlusion was confirmed by angiography. (D) The stent was sent through vessel occlusion. (E. F) DSA showed basilar artery blood recovered TICI-3 flow. (G) Thrombosis removed from a stent. CTA = computed tomography angiography.

On day 7, the patient presented to the hospital again for sudden dizziness and limb numbness accompanied by nausea and vomiting. He showed neurologic deficits in exam-muscle strength in the left limb; the knee extensors and flexors on the Lovett scale were 2, while the right limb and knee were 4. In addition, the Babinski sign was observed, and NIHSS was 8. Five hours after onset, computed tomography (CT) excluded intracerebral hemorrhage. Subsequently, the patient was treated with urokinase 1 million IU iv, after which the symptoms improved. MR imaging showed ischemic lesions in the left cerebral peduncles (Fig. 1A); computed tomography angiography showed basilar artery stenosis (Fig. 1B) on day 14, after which the symptoms reappeared. Tirofiban 5 mL/h ivvp was then given for 15 hours, and symptoms were relieved. After 3 hours, the patient suffered from sudden disturbance of consciousness. CT excluded cerebral hemorrhage and the basilar artery occlusion was considered.

Under general anesthesia, the 6F (ENVOY DA, Calle Circuito Interior Norte, Parque Industrial Salvarcar, Mexico) guiding catheter was positioned to the terminal jugular segment of the left vertebral artery via and right common femoral arterial accesses; the basilar artery occlusion was confirmed by angiography (Fig. 1C). Guided by the micro-guidewire (Synchro, Boston Scientific Place, USA), the microcatheter rebar-18 (solitaire FR, Toledo Way, Irvine, CA) was then passed through the occluded segment of the basilar artery and entered the left posterior cerebral artery. The position and length of the occluded segment were confirmed by angiography. The 4.0 mm × 20 mm stent (solitaire FR, Toledo Way) was then introduced through the microcatheter to the basilar artery occlusion terminal segment and was released (Fig. 1D). The stent was reserved for 5 min, and part of the stent was slowly preretracted, after which it was quickly removed under suction, and a thrombus was seen (Fig. 1G). Angiography confirmed TICI-3 flow in the posterior cerebral circulation (Fig. 1E), and there was no obvious displacement of the original stent position. After 20 minutes of delayed observation, angiography showed TICI-3 flow in the posterior cerebral circulation (Fig. 1F), after which the operation ended. As antiplatelet resistance was the most probably thrombotic factor in stents, the tirofiban was replaced with tirofiban, and the patient was informed about it.

The patient was followed up for half a year without recurrence of symptoms.

### 2.2. Case 2

A 44-year-old male with a history of hypertension and type-2 diabetes was admitted to the hospital due to sudden vomiting followed by a loss of consciousness that lasted for 3 hours. He showed neurologic deficits on the exam-muscle strength of left limb and knee extensors and flexors on the Lovett scale were 2, while right limb and knee were 4. Babinski sign was also observed and NIHSS was 15. MR DWI sequence showed fresh cerebral infarction in the left pontine; MR angiography showed vertebrobasilar artery occlusion and DSA showed severe stenosis of a vertebrobasilar artery in series. Left vertebrobasilar artery BDA and stent implantation were performed and the patient recovered consciousness without neurological deficits. There were no symptoms of dizziness or loss of consciousness after discharge.

**Figure 2.**
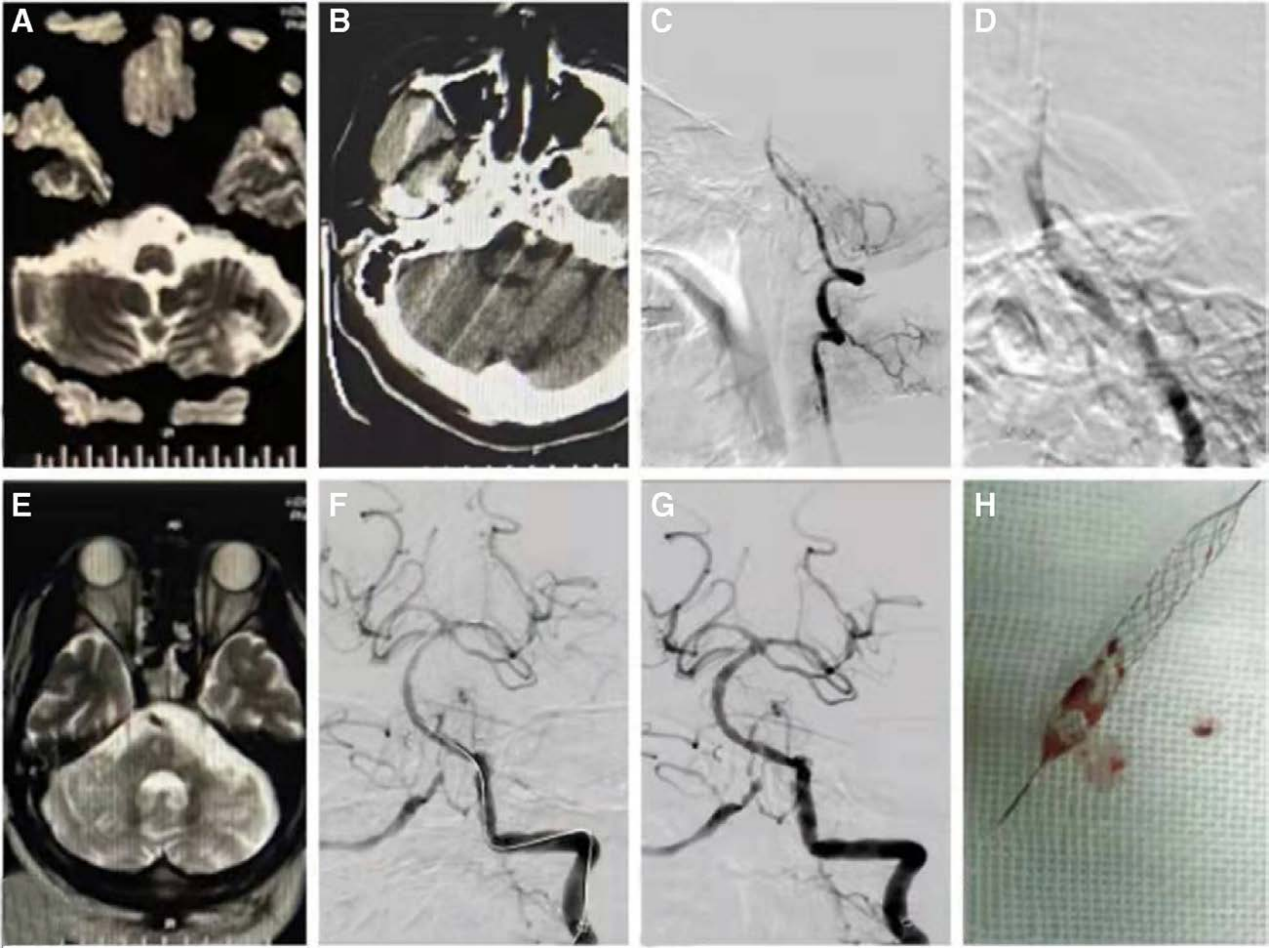
(A) DWI examination showed fresh cerebral infarction in bilateral cerebellar hemispheres and the right occipital lobe. (B) Brain CT showed hyperdense signs of the basilar artery. (C) Left vertebral artery lateral angiography showed basilar artery occlusion. (D) Lateral magnification showed that the thrombus was located in the basilar artery stent. (E) A week later, the abnormal signal of the right occipital lobe decreased. (F) The stent was sent through the blood vessel. (G) DSA showed basilar artery blood recovered TICI-3 flow. (H) The stent was withdrawn, and a thrombus was seen. CT = computed tomography.

Seven months later, he lost consciousness again. The neurologic deficits observed on the exam were worse compared to previous results, i.e., muscle strength of limb and knee extensors and flexors on the Lovett scale was 0; the Babinski sign was observed, and the NIHSS was 25. CT showed a hyperdense sign in the basilar artery without bleeding (Fig. 2B), while the MR DWI sequence showed fresh cerebral infarction in the bilateral cerebellar hemisphere and right occipital lobe (Fig. 2A). Combined with the patient’s medical history and imaging characteristics, vertebrobasilar vascular occlusion was considered.

Under general anesthesia, the 6F (ENVOY DA, Calle Circuito Interior Norte, Parque Industrial Salvarcar, Mexico) guide catheter was positioned via right common femoral arterial accesses in the V3 segment of the left vertebral artery. The basilar artery stent occlusion could be seen by angiography (Fig. 2C and D). The microcatheter (prowler-27, Fox Lane, San Jose, CA) passed the occluded segment of the basilar artery and entered the left posterior cerebral artery with (Synchro, Boston Scientific Place) the micro guidewire. The position and length of the occluded segment were confirmed by angiography; the 4.5 mm × 22 mm (Revive SE, Fermont Blvd, Fremont, CA) stent reached the terminal segment of the basilar artery occlusion via a microcatheter. It was released (Fig. 2F) and then retained for 5 minutes, after which the part of the stent was prerecovered. The stent was then retracted under standard conditions and a thrombus was seen (Fig. 2H). Angiography again showed that the posterior cerebral circulation recovered TICI-3 flow (Fig. 2G). Tirofiban 10 mL/h ivvp was then continuously given for 24 hours, followed by aspirin 100 mg po tid, and tirofiban 75 mg po qd. In case 2, the thromboelastogram was immediately checked after the operation, where the inhibition rate of AA was 86%, and the inhibition rate of ADP was 13 2%. This suggested the clopidogrel resistance; thus, tirofiban was selected to replace clopidogrel. A week later, MR imaging examination showed that the infarct area was significantly decreased (Fig. 2E), and the patient’s condition improved without deterioration of neurological function.

## 3. Discussion

Stent thrombosis is a serious complication of cerebrovascular stent implantation that requires timely treatment. The vertebrobasilar artery supplies blood to the cerebellum and brainstem. The clinical manifestations of thrombosis in a basilar artery may vary according to occlusion sites. Vertigo, nystagmus, diplopia, dysarthria, dysphagia, ataxia, crossed paralysis, and other symptoms can occur in patients with partial occlusion, while quadriplegia and consciousness disorder is more common in those with trunk occlusion.^[5]^ Factors associated with stents thrombosis include insufficient preoperative antiplatelet, antiplatelet resistance, and arterial stents that fail to adhere fully to blood vessel endophragm, resulting in excessive residual stenosis and distal hemodynamic changes or vascular endothelial injury caused by vasospasm during stent implantation.^[6,7]^

Rapid and early direct revascularization could improve the prognosis for patients with stent thrombosis.^[8]^ Here, we reported 2 cases of stent thrombosis in the vertebrobasilar artery, including their imaging and clinical findings. Case 1 was given intravenous thrombolysis, after which the symptoms improved. After the symptoms reappeared, tirofiban was given via a microinjection pump. Once tirofiban was stopped, the loss of consciousness aggravated, and Case 1 suddenly lost consciousness. Comprehensive medical history and signs were indicative of acute large vessel occlusion.

Stent retrieval and aspiration are the 2 most popular and effective approaches for large vessel occlusion.^[9]^ Aspiration thrombectomy is usually recommended first, considering it has a lower risk of potentially damaging a stent construct. Yet, when the stent embolus is located in the basal artery, the suction catheter cannot easily reach the occluded segment. In that case, stent retriever thrombectomy is used; it could reach the embolus parts more easily and determine the length of the embolus. Yet, there are certain surgical risks and technical challenges when performing stent retriever thrombectomy. The stent may be entangled with the original stent, resulting in the inability to remove the stent thrombosis or the displacement of the original stent and block of large blood vessels. In order to avoid entanglement, a closed-loop stent was used in this study, as it reduces the possibility of entanglement. On the other hand, when withdrawing the stent, we slowly pushed the microcatheter forward to prerecover the stent so as to form a conical structure at the proximal end of the stent, after which we uniformly pressed on both ends of the stent, and then quickly withdrew the stent.

## 4. Conclusion

These 2 cases of stent thrombosis (one with subacute stent thrombosis and the other one with late stent thrombosis) show the application of stent retriever thrombectomy in the treatment of intracerebral stent thrombosis is feasible. Yet, for this method, we recommend using the furthest closed-loop stent as well as the technology for prerecovering the stent when withdrawing the stent to improve the possibility of thrombus removal.

## Author contributions

**Conceptualization:** Tao Wu.

**Data curation:** Dongyi Yang.

**Formal analysis:** Yangyang Liu.

**Investigation:** Qinghai Dai, Lingfeng Shu.

**Writing – original draft:** Hang Li.

**Writing – review & editing:** Tao Wu.
